# Pilot study to assess the impact of feed-through insecticide on the sand fly density in an endemic area of zoonotic cutaneous leishmaniasis in Morocco

**DOI:** 10.1371/journal.pntd.0013767

**Published:** 2025-12-18

**Authors:** Wim Van Bortel, Raja Benkirane, Ayoub Hmidane, Temmy Sunyoto, Richard Poché, David Poché, Chafika Faraj, Zoë Dierickx, Rik Hendrickx, Tom Smekens, Guy Caljon, Epco Hasker, Kristien Cloots

**Affiliations:** 1 Department of Biomedical Sciences, Unit Entomology, Institute of Tropical Medicine: Instituut voor Tropische Geneeskunde, Antwerp, Belgium; 2 Department of Biomedical Sciences, Outbreak Research Team, Institute of Tropical Medicine: Instituut voor Tropische Geneeskunde, Antwerp, Belgium; 3 National School of Public Health (Ecole Nationale de Santé Publique) Rabat, Rabat, Morocco; 4 Health Establishment Network Service, Delegation of the Ministry of Health and Social Protection, Zagora, Morocco; 5 Department of Public Health, Unit of Mycobacterial Diseases and Neglected Tropical Diseases, Institute of Tropical Medicine: Instituut voor Tropische Geneeskunde, Antwerp, Belgium; 6 Luxembourg Operational Research and Epidemiology Support Unit, Médecins Sans Frontières, Luxembourg, Luxembourg; 7 Genesis Laboratories, Inc., Wellington, Colorado, United States of America; 8 Laboratory of Medical Entomology, National Institute of Hygiene, Rabat, Morocco; 9 Laboratory of Microbiology, Parasitology and Hygiene (LMPH), Infla-Med Centre of Excellence, University of Antwerp: Universiteit Antwerpen, Antwerp, Belgium; 10 Department of Public Health, Institute of Tropical Medicine: Instituut voor Tropische Geneeskunde, Antwerp, Belgium; International Atomic Energy Agency, AUSTRIA

## Abstract

**Background:**

Cutaneous leishmaniasis (CL) remains a significant public health concern in Morocco. *Leishmania major*, the principal cause of zoonotic CL (ZCL) in Morocco, is transmitted by the female sand fly *Phlebotomus papatasi*, with the great gerbil (*Meriones shawi*) serving as the primary reservoir host. Current control strategies predominantly rely on strychnine-treated bait targeting gerbils. Recently, insecticide-treated rodent bait has emerged as a promising alternative for ZCL control. This study evaluated the village-level impact of feed-through insecticide, involving two intervention villages and two control villages, with outcomes assessed through female sand fly density and *Leishmania* infection rates.

**Methodology/Principal Findings:**

The study was conducted in the Province of Zagora, southeastern Morocco. Three applications of fipronil-treated bait were performed during July and August 2020. Sand fly populations were monitored through nine surveys per village, with each survey spanning three consecutive nights. Two control and two intervention villages were included. In each village sand flies were collected in three epidemiological relevant locations, i.e., in the field where the application was implemented, and indoors and outdoors in the villages. Blood-fed sand flies were prioritized for analysis to increase the likelihood of detecting *Leishmania* circulation and because blood feeding sand flies are the primary target of feed-through insecticides. A negative binomial generalized linear model was used, with the number of female sand flies as the response variable. Explanatory variables included village status (intervention or control), application number (0, 1, 2, 3), and their interaction. The initial two applications of fipronil did not result in a significant reduction in sand fly density in the sampling location ‘field’ of the intervention villages (Interaction term: application 1*village type*:* IRR 0.65, CI95: 0.39—1.09; Interaction*:* application 2*village type: 0.81, CI95: 0.52—1.27). Only after application 3, a significant reduction in sand fly density was observed in the ‘field’ of the intervention villages. This reduction was observed in blood-fed sand flies only. No effect of the intervention was observed on the sand fly *Leishmania* infection rates. Furthermore, despite processing more than 3,600 sand flies, we did not detect *L. major*, the presumed cause of ZCL in this region. Yet, we detected the circulation of *L. infantum*, *L. tropica*, and *L. tarentolae* in the study area.

**Conclusions/Significance:**

This study assessing the impact of feed-through insecticide on sand fly density and *Leishmania* infection rates in an endemic ZCL area in Morocco found insufficient evidence of impact on sand fly densities to achieve epidemiological relevance. Additionally, the study revealed gaps in the understanding of the transmission cycle, as two *Leishmania* species other than *L. major* were identified as potential causes of CL in the area. These findings underscore the need for improved knowledge of the transmission dynamics to enhance control measures.

## Authors summary

Cutaneous leishmaniasis (CL) is a persistent public health issue in Morocco, largely driven by zoonotic transmission involving the parasite *Leishmania major*. The parasite is spread by female sand flies (*Phlebotomus papatasi*), with the great gerbil (*Meriones shawi*) as the primary reservoir host. Traditional control methods focus on gerbil population reduction using strychnine-treated bait, but alternative strategies are needed. This study tested the potential of feed-through insecticide using fipronil-treated bait to reduce sand fly density and *Leishmania* infection rates in southeastern Morocco. The study was conducted in two intervention and two control villages in the Zagora Province. During the summer of 2020, three bait applications were administered, with sand fly populations monitored through nine surveys per village. Blood-fed sand flies, the primary targets of the intervention, were prioritized for parasite detection. Results showed no significant reduction in sand fly densities to levels likely to affect disease transmission. Furthermore, the presence of two additional *Leishmania* species causing cutaneous Leishmania highlighted knowledge gaps in the local transmission cycle. These findings emphasize the need for improved understanding of CL dynamics to develop more effective control strategies.

## Introduction

Cutaneous leishmaniasis (CL) is a neglected tropical disease that poses a significant public health problem in Morocco. While skin lesions caused by CL can be treated, they often result in permanent scars on exposed body parts, leading to psychosocial suffering and stigma for patients [1]. Over the past 20 years, more than 80,000 CL cases have been reported in Morocco [2]. However, this figure likely underestimates the true burden, as many patients seek treatment from traditional healers or the private sector, which lead to cases being missed by official surveillance.

Three different *Leishmania* species causing CL are present in Morocco: *Leishmania major*, *Leishmania tropica*, and sporadically, *Leishmania infantum*. The anthroponotic *L. tropica* is mostly found in the central and northern regions of the country, while the zoonotic *L. major* is the primary cause of CL in the eastern and southeastern parts, though both parasites can overlap geographically [3].

*Leishmania major*, the main cause of zoonotic CL (ZCL) in Morocco, is transmitted by the female sand fly *Phlebotomus papatasi*. The primary reservoir host is the great gerbil, *Meriones shawi*, with humans serving as accidental hosts [4]. *Meriones shawi* is a semi-desert rodent well-adapted to arid environments, often found in burrow systems near agricultural areas where it thrives on vegetation [5]. *Phlebotomus papatasi* shares this habitat and frequently feeds on *M. shawi*, creating an ecological link that facilitates the transmission of *Leishmania* between wildlife and human-associated environments. Besides case management, disease control efforts for ZCL in Morocco primarily focus on vector and reservoir control. Historically, environmental management has been central to control activities, emphasizing improved waste disposal and plastering walls to reduce vector and reservoir abundance in and around homes [6,7].

Studies have investigated the effectiveness of insecticide-treated bed nets (ITNs) and indoor residual spraying (IRS) on vector density. These studies suggested a non-significant reduction in sand fly populations with ITNs and a potential reduction in ZCL cases with IRS [8–10]. Both ITNs and IRS are mentioned in the 2010 National Guidelines, with IRS only in case of *L. tropica* transmission, though they are not systematically implemented [6]. Following a ZCL outbreak in 2010 that mainly affected the Errachidia, Tinghir, and Zagora provinces in the Draa-Tafilalet region, the Ministry of Health joined forces with the Ministry of Agriculture to use strychnine-treated bait for crop pest control, which targeted *Meriones* [11]. Strychnine is a neurotoxin causing death by asphyxia. In Errachidia province, which was severely affected by the 2010 outbreak, strychnine-poisoned bait was used extensively for reservoir control. Although the intervention was perceived as beneficial, its true impact is unclear, and significant concerns were raised regarding its potential environmental and human hazards [11].

Insecticide-treated rodent bait has surfaced over the last years as a promising alternative for ZCL control. Feeding rodents with bait containing an insecticide result in the uptake and circulation of a systemic insecticide in the blood, killing not only the sand fly vectors feeding on the rodent, but also the larvae feeding on the rodent faeces [12]. The first insecticide used in this approach was imidacloprid, which proved to be effective for the reduction of fleas and ticks in ground squirrels and black prairie dogs, both reservoirs of human plague [13,14]. More recently, fipronil has emerged as a promising compound for use as a systemic insecticide. Fipronil is a broad-spectrum phenylpyrozol that blocks the ligand-gated ion channel of the GABA receptor and glutimate-gated chloride channels, leading to central nervous system toxicity in arthropods [15]. Fipronil has demonstrated superior performance, relative to other candidate compounds, in controlling fleas and ticks [16]. Fipronil has demonstrated effectiveness at low doses, with a nominal concentration of 0.005% fipronil being highly efficacious in controlling fleas infesting black-tailed prairie dogs [17,18], ticks infesting white-footed mice [19,20], and 0.0025% fipronil has proven efficacious in controlling ticks on white-tailed deer [21]. For sand flies, several candidate insecticides were tested, and fipronil was found to have the best performance, with an 80% reduction in *Ph. papatasi* up to six weeks after a single application of a bait containing 0.005% fipronil under field conditions in Tunisia [22]. Also the impact of orally-administered fipronil on *Phlebotomus argentipes* showed promising results [23,24]. Unlike strychnine-treated baits, these insecticides do not pose a fatal risk to mammals or humans at operational dosages [25,26].

This study aimed to assess the effectiveness of fipronil-treated rodent baits in reducing the abundance of sand flies, focusing on *Ph. papatasi* as the primary vector species for ZCL in Morocco. Unlike previous studies, we evaluated the impact of the feed-through insecticide at the village level under semi-operational conditions, involving two intervention and two control villages. The impact of the treatment was assessed based on female sand fly density and *Leishmania* infection rates in engorged sand flies. Female sand flies serve as an indicator for sand fly control because only they take blood meals and can transmit CL.

## Methods

### Ethics

The study protocol was approved by the Ethics committee of the National School of Public Health (reference number 53/19; approved on 14/03/2019), Morocco. Written informed consent was obtained from all household owners who provided access to their property for the entomological surveys. Aggregated data of the CL cases at village level were obtained from the routine surveillance system.

### Study area

The study was conducted in the Province of Zagora, located in the Draa-Tafilalet region of southeastern Morocco. Zagora is located at an altitude of 700 m asl. The region is characterised by a hot dry climate with mean daily temperatures ranging from 17 – 42 °C depending on the season, with an annual rainfall of 16 mm, and about 41 rainy days per year. This region relies heavily on agriculture, with date palm cultivation being a primary source of livelihood for local communities. Other crops, including grains, vegetables, and fruits, are also grown. The Draa-Tafilalet region features diverse ecosystems, ranging from arid desert plains to green oases sustained by the Draa River.

**Fig 1:**
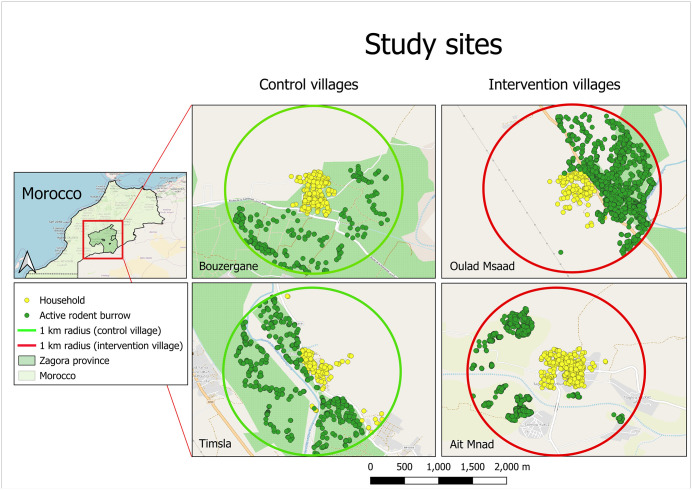
The location of the study villages in the Province of Zagora south-eastern Morocco and the location of the active *Meriones shawi* burrows identified in the four villages at the start of the study (green dots), in relation to the location of the human habitations (yellow dots). Situation as of May 2020. The baseline administrative map is derived from https://diva-gis.org/data.html and the basemap is OpenStreetMap https://www.openstreetmap.org/#map=8/50.730/5.367. The boundaries shown on the map do not imply any judgment or position on the legal status of any country or territory.

Based on the CL case load from the year prior to the study (2019) – data obtained from the routine surveillance system – all villages within Zagora province were ranked from high to low. Considering accessibility of each village, as well as population size, ecology of the village area and acceptability by the village representative to collaborate in the study, four villages were selected and randomly categorized as either intervention or control village. Aid Mnad and Oulad M’Saad were designated as intervention villages, while Timsla and Bouzergane were chosen as control villages. The exact number of reported CL cases per study village is provided in S1 Table. The geographical locations of the selected villages are illustrated in Fig 1. All fieldwork was conducted between 15/06/2020 and 18/11/2020, within a 1 km radius around the centre of each village. The centre of the village was determined by calculating the centroid of the polygon determined by the households belonging to the village, using qGIS software.

### Intervention and study design

Before the intervention, active rodents burrows in a radius of 1 km around each village were mapped using ODK data collection platform (https://getodk.org/index.html) in order to facilitate the application of the fipronil-treated bait (Fig 1). The rodent grain bait, containing a nominal concentration of 0.005% fipronil (Genesis Laboratories, Inc. Wellington, Colorado, United States of America) (50 mg/kg), was prepared locally, as described by Poché *et al.* [12,17]. In the two intervention villages, Aid Mnad and Oulad M’Saad, the bait was applied within maximum 1 meter outside of each identified rodent burrow using approximately 50 g of fipronil bait per burrow. A picture was taken from each application of fipronil-treated bait (S1 Fig), georeferenced with Global Positioning System (GPS) coordinates, for monitoring purposes. Three applications of fipronil-treated bait were done: on 9-10 July 2020; on 18-19 July 2020; and on 18-19 August 2020. The concentration of the fipronil-bait used was verified post-factum in order to confirm that the dosage was not a cause of negative results.

### Sand fly collections and processing

Sand flies were collected in the four study villages through nine surveys in each village, with each survey lasting three consecutive nights. The first six surveys were conducted biweekly, and from September 2020 onwards, the surveys were carried out monthly. Each night, 30 CDC miniature light traps (Model 2836 BQ; BioQuip Products, Rancho Dominguez, CA 90220, USA) were deployed per village, including 10 traps in the field (referred to as field traps), 10 traps inside houses where people sleep (referred to as indoor traps), and 10 traps in the animal shelters of the same houses used for indoor collections (referred to as outdoor traps).

The ‘field’ sampling location was chosen because it was the site of the actual intervention, where the greatest effect of the feed-through treatment was expected. Human infection is presumed to occur peri-domestically, which is why we also evaluated the impact on sand fly density outdoors in the villages in the animal sheds and indoors where people sleep during the night.

After each collection night, the collection bags were brought to the field laboratory where sand flies were separated from other insects. The sand flies were then sorted by sex, and the females were further classified as blood-fed (i.e., engorged sand fly with red swollen abdomen), non-blood-fed (i.e., non-engorged sandfly without blood meal seen in the abdomen), or gravid. The sorted specimens were transferred into 1.5 ml Eppendorf tubes containing 97% ethanol and labelled accordingly. At the Institut National d’Hygiène (INH), males and non-blood-fed females were identified based on the morphology of the pharynges and/or the male genitalia or female spermathecae, as described by Faraj and Himmi [27]. Prior to morphological species identification, sand flies were cleared in Marc-André solution (40 g chloral hydrate, 30 ml acetic acid, 30 ml distilled water) [28]. Blood fed and gravid female sand flies were transported to the Institute of Tropical Medicine (ITM), Antwerp, Belgium, for subsequent molecular analyses. Blood-fed female sand flies were processed for *Leishmania* spp. detection. We focussed on blood-fed sand flies to assess the circulation of the *Leishmania* parasites to increase the chances of finding the *Leishmania* parasites and also because the first target of the feed-through insecticide are blood feeding sand flies.

### DNA extraction

DNA extraction was performed as described by Pareyn et al. [29]. Briefly, individual sand flies were incubated overnight at 37°C on a shaker at 150 revolutions per minute in 50 μL of extraction buffer (10 mM Tris-HCl pH 8, 10 mM EDTA, 0.1% SDS, 150 mM NaCl) with 0.5 μL of proteinase K (200 μg/ml). Following incubation, 25 μL of ultrapure water was added, and the sample was heated for 5 minutes at 95°C. Negative extraction controls, consisting of ultrapure water, were included.

The individual sand fly extracts were cross-pooled in duplicate following the method described by Pareyn et al. [29]. Cross-pooling allowed each sample to be tested twice, enabling the identification of individual sand flies in positive pools for subsequent molecular identification of both the sand fly species and the *Leishmania* species (see section “*Leishmania* detection and identification in sand flies”).

To purify the pools, 1/10th volume of 3M NaOAc (pH 5.6) and 2 volumes of -20°C 100% ethanol were added to each pool, followed by overnight incubation at -20°C. The samples were centrifuged for 15 minutes at 16,100 RCF at 4°C, and the supernatant was removed. The pellet was washed with 500 μL of 70% ethanol (chilled at -20°C) and centrifuged again. The pellet was then dried in a heating block at 50°C. Finally, the DNA was resuspended in 20 μL of ultrapure water.

### Leishmania detection and identification *in* sand flies

Pools were tested with a one-step RNA real-time quantitative PCR (one-step RT-qPCR) assay targeting the conserved and highly expressed spliced-leader (SL) mini-exon sequence [30]. The 39-bp sequence of SL-RNA is strongly conserved between various old and new world *Leishmania* species including *L. major*, *L. tropica*, *L. infantum*, and *L. donovani* and showed excellent results in the detection of *Leishmania* spp. in sand fly species [31].

A 20-μL reaction mixture contained 10 μL 2 × SensiFAST SYBR Hi-ROX One-Step mix (Bioline), 0.8 μL forward (5’ -AACTAACGCTATATA AGTAT- 3’) and reverse (5’-CAATAAAGTACAGAAACTG- 3’) primer (0.4 µM), 0.2 μL of reverse transcriptase, 0.4 μL of RNase inhibitor, 4 μL of RNA (1/5 diluted) and 3.8 μL of PCR-grade water. PCR reactions were performed on a StepOnePlus real-time PCR system (Applied Biosystems) using a 10-minute reverse transcription at 45°C followed by a 2-minute activation at 95°C and 40 amplification cycles under optimized conditions (15 sec at 95°C, 15 sec at 56°C and 15 sec at 72°C). PCR was followed by a melt curve analysis: 95°C for 15 seconds, 45°C for 1 minute, 95°C for 15 seconds, step and hold with 0.3°C increments. Melting temperatures of 66.9 ± 0.5°C were evaluated as specific amplification products in the SL-RNA qPCR reaction. Subsequently, individual sand fly specimen from the positive pools were tested by the same SL-RNA assay to confirm the results after which *Leishmania* species identification was performed by sequencing part of the internal transcribed spacer 1 (ITS-1) region [30,32]. Samples which were positive for SL-RNA but negative for ITS-1 were subjected to an ITS-1 repeat PCR to increase sensitivity. A 25 µL PCR reaction mixture contained 12.5 µL -of 2x GoTaq G2 Master Mix (Promega), 5 µL sample (1/5 diluted), 1.25 µL of forward (5’-CTGGATCATTTTCCGATG-3’) and reverse (5’-TGATACCACTTATCGCACTT-3’) primer (0.5 µM) and 5 µL of PCR-grade water. Samples were tested on a Bio-Rad T100 thermal cycler using a 5-minute activation step at 95°C, 35 amplification cycles under optimized conditions (30 sec at 95°C, 30 sec at 53°C and 30 sec at 72°C) and a 10-minute final elongation at 72°C. PCR products were checked by 1% agarose gel electrophoresis and purified using a QIAquick PCR Purification Kit (Qiagen) after which negative samples were subjected to a nested PCR reaction using the same conditions as described above. PCR samples were again checked by 1% agarose gel electrophoresis and purified with a QIAquick PCR Purification Kit after which all positive samples were sent for Sanger sequencing on an Applied Biosystems 3730XL DNA Analyzer at the Neuromics Support Facility of the University of Antwerp. *Leishmania* species identification was analysed using SnapGene and NCBI BLAST.

### Molecular identification *of* sand flies

The sand fly specimens positive for *Leishmania* spp. were identified by DNA barcoding based on the amplification of the mitochondrial cytochrome c oxidase subunit I (COI) gene. A 25 µL PCR reaction mixture contained 12.5 µL 2X of GoTaq G2 Master Mix (Promega), 1 µL sample and 0.4 µL (0.4 µM) forward LCO1490 (5’-GGTCAACAAATCATAAAGATATTGG-3’) and reverse HCO2198 (5’- TAAACT TCAGGGTGACCAAAAAATCA-3’) primer and 10.7 μL of PCR-grade water [33–35]. Samples were tested on a Biometra TAdvanced thermal cycler using a 2-minute activation step at 95°C, 40 amplification cycles under optimized conditions (30 sec at 95°C, 45 sec at 50°C and 60 sec at 72°C) and a 10-minute final elongation at 72°C [36]. PCR products were checked by 2% agarose gel electrophoresis after which they were sent for Sanger sequencing at Baseclear B.V. Sequences were verified and blasted against NCBI and BOLD using Genious. The species identification engine of BOLD was used with the species-level barcode records option to find the closest matching reference sequence.

### Data analysis

To evaluate the impact of the feed-through bait on female sand fly density, we fitted a negative binomial generalized linear mixed model (GLMM) with the number of female sand flies as response variable. The status of the village, i.e., intervention or control, and the application number (0, 1, 2, 3), as well as the interaction between both were included as explanatory variables, allowing us to estimate their associations with the response variables as Incidence Rate Ratios (IRR) showing the relative difference in sand fly density measured as number of sand flies per trap-night. Random effects were added for the trap identification number, unique for each trap-location, to account for systematic and random natural variation in sand fly counts between trap locations. Models were built for each sampling location (field, indoor, outdoor) separately. Similar models were built for each sand fly species where possible. The R package lme4 was used to fit the models [37].

The prevalences and their 95% confidence interval (95 CI) of the *Leishmania* spp. infection in blood-fed sand flies were obtained by a maximum likelihood estimate using the R package PoolTestR [38]. The effect of the intervention on the *Leishmania* prevalence in blood-fed sand flies was tested using a GLMM with binomial distribution with the same parameters as the model on female sand fly density, with the *Leishmania* spp. positivity rate based on the SL-RNA assay as response variable.

## Results

### Fipronil application

Active rodent burrows were mapped before the intervention. *Meriones shawi* burrows were identified in close proximity of the villages Bouzergane (active burrows, n = 459), Oulad Msaad (active burrows, n = 2050), and Timsla (active burrows, n = 780) which are situated in the oasis of greenery and date palms along the Draa River. In Ait Mnad burrows (active burrows, n = 2568) were found at a distance of at least 800 m from the village (Fig 1).

The post-factum analysis of the concentration of the fipronil-bait showed an effective fipronil dosage of 0.00536%, close to the target dose of 0.0055%.

### Sand fly collection

**Fig 2:**
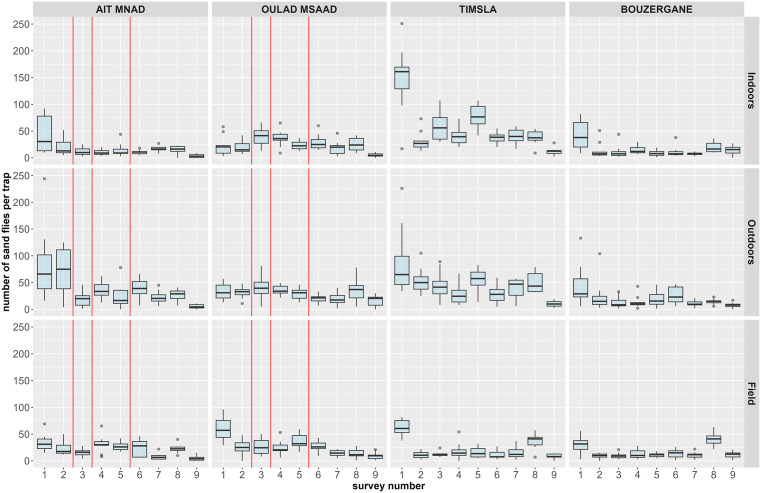
Number of collected sand flies (males and females combined) presented as boxplots providing an overview of the medians and interquartile ranges of the counts per trap over three nights by village, collection place and survey. The red vertical lines indicate the application of fipronil bait in the intervention villages Ait Mnad and Oulad Msaad. Timsla and Bouzergane were the control villages.

In total 29,232 sand flies (male and female combined) were collected for 3,240 trap-nights. Most sand flies were collected in Timsla with a high number of individuals in survey 1 and indoors (Table 1 and Fig 2). In Ait Mnad most sand flies were collected outdoors in the animal shelters. In Oulad Msaad and Bouzergane the number of sand flies per collection place was similar (Table 1).

**Table 1:** Total number and row percentage of sand flies (male and female combined) collected per village and place of collection.

Study village	Collection place			
	**Indoors**	**Outdoors**	**Field**	**Total**
**Ait Mnad (intervention)**	1463 (22%)	3265 (49%)	1891 (29%)	6619
**Oulad Msaad (intervention)**	2192 (31%)	2621 (36%)	2372 (33%)	7185
**Timsla (control)**	4872 (45%)	3977 (37%)	1913 (18%)	10762
**Bouzergane (control)**	1439 (31%)	1737 (37%)	1490 (32%)	4666

**Fig 3:**
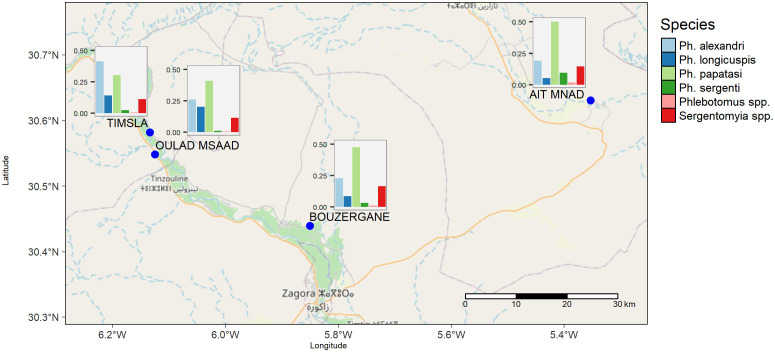
Overview of the proportional presence of sand fly species in the four study villages. *Phlebotomus* spp include *Ph. africana, Ph. bergeroti, Ph. chabaudi, Ph. chadlii, Ph. kazeruni, Ph. longeroni*. Sergentomyia spp. include *S. clydei, S. dreyfussi, S. fallax, S. lewisi, S. minuta, and S. schwetzi*. The basemap is obtained from OpenStreetMap (https://www.openstreetmap.org/#map=7/32.059/-8.405) sources using the read_osm function in **R.**

Ten different *Phlebotomus* sand fly species were morphologically identified. The most abundant species were *Phlebotomus alexandri, Phlebotomus longicuspis, Ph. papatasi,* and *Phlebotomus sergenti* (Fig 3). In Ait Mnad, Bouzergane and Oulad Msaad *Ph. papatasi* was the most abundant sand fly species followed by *Ph. alexandri*. In Timsla, *Ph. alexandri* was more abundant than *Ph. papatasi*. In all study villages these four sand fly species were caught in all sampling locations, i.e., in the field, indoors and outdoors. The other six Phlebotomine species, *Phlebotomus africana, Phlebotomus bergeroti, Phlebotomus chabaudi, Phlebotomus chadlii, Phlebotomus kazeruni,* and *Phlebotomus longeroni*, only represented one percent of the *Phlebotomus* spp. collection (Fig 3).

The species belonging to the *Sergentomyia* genus presented 11–17% of the collection (Fig 3) with following species morphologically identified: *Sergentomyia clydei, Sergentomyia dreyfussi, Sergentomyia fallax, Sergentomyia lewisi, Sergentomyia minuta,* and *Sergentomyia schwetzi.*

### Impact *of the* intervention *on* female sand fly density

The evaluation of the impact of the fipronil feed-through application was done on female sand fly collections of surveys 2–7. Survey 2 was conducted before the start of the application in the intervention villages, and up to survey 7 collections were done biweekly with the same interval between the collections – from survey 8 collections were monthly. The model used survey 2 (pre-intervention) and control villages as baseline references.

**Fig 4:**
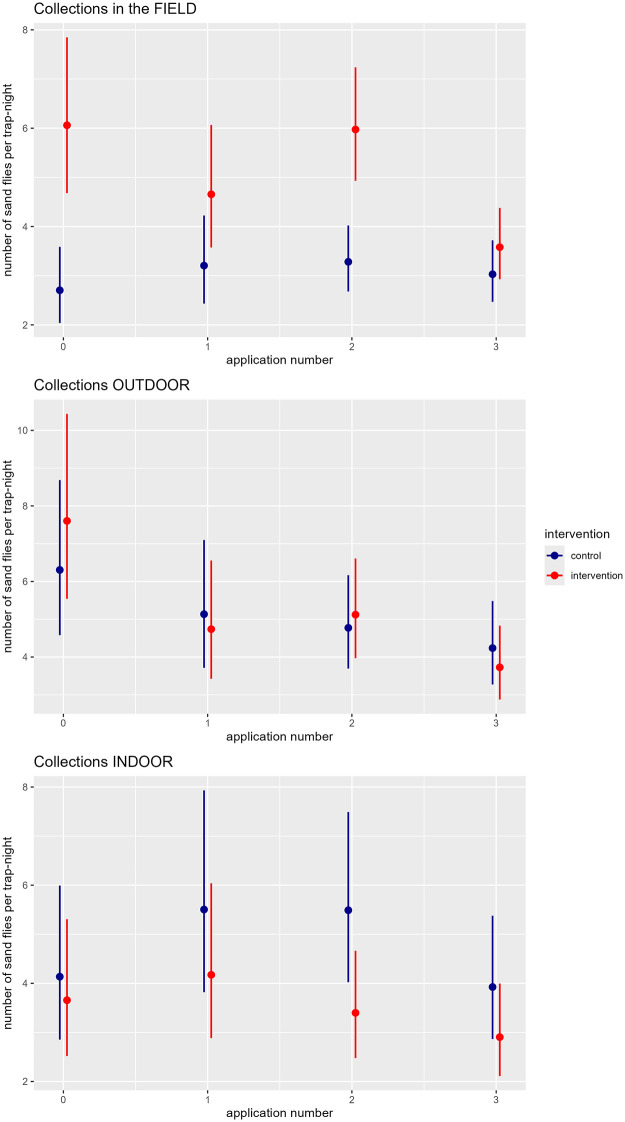
Predicted female sand fly counts and their 95% confidence intervals in function of the application number and the village type (control, intervention) by sampling location (field, outdoor, and indoor) based on the GLMM model. Application 0 refers to the baseline, i.e., before any intervention.

At baseline (survey 2), we collected an average of 2.7 female sand flies per trap-night in the sampling location ‘field’ of the control villages (95 CI: 2.0 – 3.6). At baseline (survey 2), 2.24 (95 CI: 1.53 – 3.29, p < 0.001) times more female sand fly were caught per trap-night in the location ‘field’ of the intervention villages compared to the control villages (Fig 4). Outdoors the sand fly density at baseline, i.e., in survey 2 in the control villages, was 6.31 sand flies per trap-night. Indoors this was 4.14 sand flies per trap-night. The baseline (survey 2) collections indoors (IRR: 0.88; 95 CI:0.52 – 1.51; p = 0.651) and outdoors (IRR 1.21; 95 CI: 0.77 – 1.90; p = 0.420) were similar in both intervention and control villages (Fig 4).

The initial two applications of fipronil did not result in a significant reduction in sand fly density in the sampling location ‘field’ of the intervention villages (Interaction: application 1 *village type*:* 0.65, CI95: 0.39—1.09; Interaction*:* application 2*village type: 0.81, CI95: 0.52—1.27) (Table 2 and Fig 4). However, after application 3, a significant reduction in sand fly density was observed in the field collections of the intervention villages compared to pre-intervention collections, which was not observed in the control villages. Specifically, the sand fly density in the field collections decreased from 6.06 (CI95: 4.67 – 7.86) per trap-night before the intervention to 3.03 (CI95: 2.47 – 3.72) after application 3 (Table 2 and Fig 4). Further analysis revealed that this reduction was observed in blood-fed sand flies only (S2 Fig).

**Table 2:** The effect of village type (control or intervention), application number (0, 1, 2 or 3) and their interaction on the female sand fly counts based on a GLMM with negative binomial distribution. 95 CI = 95% confidence interval.

Predictors	Sampling location								
	**FIELD**			**OUTDOOR**			**INDOOR**		
	**Incidence Rate Ratio**	**95 CI**	**p**	**Incidence Rate Ratio**	**95 CI**	**p**	**Incidence Rate Ratio**	**95 CI**	**p**
application 1	1.19	0.81 – 1.73	0.378	0.81	0.55 – 1.21	0.314	1.33	0.89 – 1.99	0.163
application 2	1.21	0.87 – 1.69	0.248	0.76	0.54 – 1.07	0.113	1.33	0.94 – 1.89	0.112
application 3	1.12	0.81 – 1.56	0.500	0.67	0.47 – 0.95	**0.025**	0.95	0.67 – 1.35	0.771
Intervention village	2.24	1.53 – 3.29	**<0.001**	1.21	0.77 – 1.90	0.420	0.88	0.52 – 1.51	0.651
appl 1 x Intervention	0.65	0.39 – 1.09	0.100	0.77	0.43 – 1.35	0.355	0.86	0.49 – 1.51	0.596
appl 2 x Intervention	0.81	0.52 – 1.27	0.361	0.89	0.55 – 1.45	0.639	0.70	0.43 – 1.15	0.159
appl 3 x Intervention	0.53	0.34 – 0.83	**0.005**	0.73	0.45 – 1.19	0.207	0.84	0.51 – 1.38	0.485

In the outdoor collections no impact of the intervention was observed. Likewise, the application had no impact on the sand flies collected indoors.

Considering the four most abundant sand fly species separately (i.e., *Ph. alexandri, Ph. longicuspis, Ph. papatasi,* and *Ph. sergenti*) the models produced only single fits. Based on the simplified models, removing the random effect from the model, the feed-through insecticide had no impact on the density of the species *Ph. papatasi, Ph. alexandri* nor *Ph. longicuspis* (S2 Table). The models of *P. sergenti* did not produce meaningful results.

### Circulation *of* Leishmania spp *in the* study villages based *on* detection *in* blood-fed sand flies

The *Leishmania* spp. prevalence in blood-fed sand flies ranged from zero to 2.17%. *Leishmania* infected sand flies were primarily found outdoors and in the field (Table 3). The impact of the intervention on the *Leishmania* prevalence could not be tested in the sampling locations ‘field’ and ‘indoor’ because in some of the categories all samples were negative. In the intervention villages, for outdoors there was a tendency towards a lower *Leishmania* spp infection rate in blood-fed sand flies after the intervention (odds ratio 0.31, CI95 0.04—2.50), but this was not significant (*p* = 0.275) (S3 Table).

**Table 3:** Overview of the *Leishmania* spp. prevalence in blood-fed sand flies and *Leishmania* species detection in *Leishmania*-specific SL-RNA positive samples and the identification of the *Leishmania* spp. based on the ITS-1 sequencing.

Sampling place	Village	Number tested	Number of SL-RNA positive	Percentage positive	CI95	*Leishmania* species identification based on ITS-1 sequencing in *Leishmania*-specific SL-RNA positive samples				
						*Leishmania infantum* ^*1*^	*Leishmania tarentolae*	*Leishmania tropica*	Unidentified species	Negative^2^
Field	Ait Mnad	476	3	0.63	0.16-1.63	1				2
	Bouzergane	260	2	0.77	0.13-2.36			1		1
	Oulad Msaad	599	5	0.83	0.3-1.79	1	1		1	2
	Timsla	159	1	0.63	0.04-2.74					1
Outdoor	Ait Mnad	276	6	2.17	0.87-4.36			4		2
	Bouzergane	292	6	2.05	0.82-4.12				1	5
	Oulad Msaad	288	3	1.04	0.26-2.68		3			
	Timsla	319	3	0.94	0.23-2.42			1		2
Indoor	Ait Mnad	124	0	0	0-1.54					
	Bouzergane	236	3	1.27	0.32-3.26			1	1	1
	Oulad Msaad	324	0	0	0-0.59					
	Timsla	305	3	0.98	0.25-2.53				1	2

^1^*Leishmania infantum* is *Leishmania infantum/donovani* as the ITS-1 sequencing does not allow the distinction between these two species; ²The ITS-1 PCR-sequencing is less sensitive than the SL-rRNA assays.

The individual sand flies positive for the SL-RNA assays were amplified using the ITS-1 PCR and sequences. Not all SL-RNA positive samples could be amplified as the ITS-1 PCR is less sensitive. This reduced sensitivity can be attributed to the fact that there are fewer ITS-1 molecules per *Leishmania* parasite compared to SL-RNA molecules (Table 3) [39]. Based on the sequencing results we found two sand fly specimens positive for *Leishmania infantum/donovani* both detected in the ‘field’; one in Ait Mnad and one in Oulad Msaad. In both cases the parasites were detected in blood-fed *Sergentomyia* species. In seven blood-fed sand flies we found *L. tropica* which was found in the ‘field’, ‘outdoors’ and ‘indoors’. This *Leishmania* species was found in blood-fed *Sergentomyia* species (n = 4) and in *Ph. sergenti* (n = 3). Finally, we also detected *Leishmania tarentolae* in the ‘field’ and ‘outdoors’. This parasite species was found in blood-fed *Sergentomyia* species.

## Discussion

This study aimed to assess the impact of the feed-through insecticide fipronil on the density of female sand flies in a focus of ZCL caused by *L. major* under semi-operational conditions. We found no evidence of reduced vector density indoors or outdoors after three applications in the intervention villages. At the field sampling locations the first and second application did not result in a reduction in sand fly density. Only after application 3 we observed a significant reduction in sand fly counts. This reduction was only evident in blood-fed sand flies. Additionally, there was no observed effect on the circulation of *Leishmania* spp. based on the infection rate in blood-fed sand flies caught outdoors. The data of the other sampling localities did not allow for a formal analysis of the intervention effect on *Leishmania* spp. circulation.

In contrast, in laboratory settings, fipronil has demonstrated efficacy, showing a 100% reduction in *Ph. argentipes* three weeks post-treatment in India and significant reductions in blood-fed *Ph. papatasi* in Israel up to 9–15 days post-treatment, depending on the treated animal reservoir [23,40]. A field trial in Tunisia showed an 80% reduction in *Ph. papatasi* six weeks post-treatment, with sand flies caught using sticky traps at the entrance of burrows [22]. Furthermore, a field trial in Israel reported a significant reduction in blood-fed female *Ph. papatasi* inside nets covering the burrows; however, no such reduction was found outside the enclosure [41]. Conversely, results from a field trial in Kazakhstan were inconsistent. While a 100% reduction in gravid *Phlebotomus mongolensis* sand flies was observed after feeding on great gerbils, there was no noticeable reduction in overall (gravid and non-gravid female) sand fly numbers three weeks post-treatment based on collections using CDC light traps positioned 1 meter from the treated burrows [12].

Unlike previous studies, we investigated the effect of the feed-through insecticide fipronil in a semi-operational setting. The insecticide was applied within a 1 km radius around villages near pre-identified active rodent burrows. We assessed the impact in three epidemiologically relevant sampling locations. We expected greatest effect in the field, as this was the actual intervention site where *M. shawi* resides. This location is where the enzootic cycle occurs and where sand flies are expected to feed on the reservoir hosts. Since human infection is thought to happen peri-domestically, we also evaluated the impact on sand fly populations both outdoors in the villages and indoors where people sleep. Instead of sampling near the burrows as done in prior studies [22,41], we positioned CDC light traps throughout the field independent of their distance to the nearest burrow to measure sand fly density in this location. We believe our approach offers a more realistic and epidemiologically relevant assessment of the impact of the feed-through insecticide application on human infection. As we did not observe any reduction in sand fly density peri-domestically we assume that the application as implemented will not result in a positive epidemiological impact. Additionally, the impact on the enzootic cycle is expected to be limited as we observed only a reduction in blood-fed sand fly counts after the third application. Therefore, if three applications are necessary to see a reduction in sand flies, it raises questions about whether this approach would sustainably reduce *Leishmania* transmission and whether it is logistically feasible.

Several reasons can explain why a clear reduction, as seen in the other studies, was not observed. First, following the 2018 outbreak, the national program intensified its control measures. These efforts included using strychnine rodenticides in agricultural areas and Brodifacoum in peri-domestic areas from November to February. Additionally, deltamethrin insecticide was applied peri-domestically in villages from May to August after cleaning initiatives aimed at removing garbage accumulation points. The intensification of these activities since 2018 might have reduced the animal reservoirs and vectors to such a low level that the effect of fipronil was not noticeable. However, control measures were implemented in all study villages, and two villages without fipronil application were included to account for the potential impact of the control measures implemented by the national programme. Moreover, an inventory of active rodent burrows before the study showed that *M. shawi* was still abundant and active in the study areas. Second, in a 2019 pre-intervention pilot study in Errachidia province, wildlife cameras confirmed that *M. shawi* consumed the bait (Poché, personal communication), but no such confirmation was possible during the current pilot study. Nevertheless, the observed impact on blood-fed sand flies and the fact that the bait was no longer present before each new application, as observed by the field team, suggest that the insecticide-treated bait was indeed consumed. Third, the timing of the fipronil application was based on the seasonality of sand flies in southeastern Morocco, aligning with the peak sand fly density [42], rather than the seasonal activity of rodents. Subsequent treatments were administered at two and four-week intervals to ensure sufficient insecticide presence in the treatment villages, but clear guidance was missing. Fourth, insecticide resistance is a concern when applying compounds in the field. Faraj et al [43] found no resistance to lambdacyhalothrin, DDT, and malathion in *Ph. sergenti* and *Ph. papatasi* in seven study villages throughout Morocco. However, research on fipronil resistance in sand flies is lacking, but there is little direct evidence to suggest that resistance to fipronil occurs. Fifth, gaps in our knowledge regarding sand fly species in this region should be taken into consideration. While *M. shawi* has been implicated as a ZCL reservoir, the specific blood meal preferences of the sand fly species in this region are not entirely known, and in specific situations dead-end hosts can make up a sizeable portion of the bloodmeals taken. For example, on the Indian subcontinent, *L. donovani has* been largely believed to be transmitted anthroponotically [44]. However, village bovids encompass a sizable proportion of the blood meals taken by the vector *P. argentipes* [45,46]. The presence of livestock in the current region suggests this could be a possibility. Additionally, while fipronil is excreted in animal faeces and lethal to larvae when consumed [23,24,47], it is unclear what proportion of larvae are feeding on rodent faeces, as there is a deficit of empirical evidence regarding oviposition site selection by phlebotomine sand flies [48]. Previous ecological modelling has suggested that these two factors greatly influence the effectiveness of systemic insecticide control in *P. argentipes* at the village level [49]. More information regarding the ecology of sand flies in this region would be extremely helpful in the development of management strategies.

The xenomonitoring was based on a two-step approach by using first a very sensitive universal *Leishmania* spp detection assay after which we applied ITS1 sequencing for the species identification. This approach revealed the circulation of *L. infantum*, *L. tropica*, and *L. tarentolae* in the study area. Despite processing more than 3,600 sand flies, we did not detect *L. major*, the presumed cause of ZCL in this region. *Leishmania infantum* is predominantly found in northern Morocco, but it is spreading to new areas within the country [3,50,51]. *Phlebotomus longicuspis*, one of the known vector species of *L. infantum* in Morocco, was found in all four study villages, along with the host reservoir, i.e., dogs and stray dogs. Hence, the vector-borne transmission of this parasite in southeastern Morocco is likely. Additionally, in central Morocco, DNA of this *Leishmania* species was found in three rodent species, i.e., *Mus musculus*, *Rattus norvegicus*, and *Rattus rattus*, leading Echchakery et al. (2017) to conclude that the possible involvement of rodents in *L. infantum* cycles should not be excluded. *Leishmania tropica* is considered an anthroponosis, although non-human reservoirs are often suggested [51]. This parasite, along with its main vector, *Ph. sergenti*, is widespread in Morocco primarily across central regions from the Atlantic to the Mediterranean [52,53]. We found both *L. tropica* and *Ph. sergenti* in the four study villages, both indoors and outdoors, close to humans. Hence, this transmission cycle should be considered as a cause of CL in the study area. Finally, *L. tarentolae* is a saurian-associated parasite circulating in the Mediterranean region. The sand fly vector associated with this parasite is *Sergentomyia minuta*, although it was also detected in *Phlebotomus perniciosus* [54]. While *L. tarentolae* is considered a non-pathogenic protozoan of reptiles, some strains of *L. tarentolae* appear to be transiently infective to dogs [54–56]. These findings suggest that the distribution of *Leishmania* species is more dynamic and evolving than previously thought, with evidence of two additional human-pathogenic species circulating in an area formerly considered endemic exclusively for *L. major* [53]. Regular *Leishmania* species identification on a subsample of patients could be considered to monitor distribution of circulating species and adapt public health interventions to a changing reality.

Based on the current pilot study assessing the impact of feed-through insecticide on the sand fly density and infection rate in an endemic area of ZCL in Morocco, we conclude that the intervention did not demonstrate sufficient impact on sand fly densities to generate an epidemiological impact. In addition, our findings highlight the need to monitor *L. major* infection among targeted animals when strychnine or other mammal-targeting interventions are implemented, in order to regularly reassess the assumptions made on *L. major* circulation, as distribution of pathogens such as *Leishmania* should be considered a dynamic phenomenon. Further, we found that there is insufficient knowledge of the actual transmission cycle as we identified two *Leishmania* species other than *L. major* which could be the cause of CL in this area. The detection of *L. infantum* and *L. tropica* underscores a significant gap in knowledge regarding local transmission dynamics and highlights the need for species-specific control strategies. For instance, the national leishmaniasis control programme recommends indoor residual spraying (IRS) when *L. tropica* incidence exceeds 12 cases per 1,000 individuals. A more comprehensive understanding of the transmission cycle—including vector and reservoir ecology and the geographic distribution of *Leishmania* species—is therefore essential for the development of more effective and targeted interventions.

## Supporting information

S1 Fig Georeferenced pictures taken by the field teams during bait application. (PDF)

S2 Fig Predicted female sand fly density and their 95% confidence intervals (CI) in function of the type of village (control versus intervention) and application number (0, 1, 2, 3) by the sampling location (field, outdoor, indoor) and physiological status of the sand flies (fed, non-fed) based on the GLMM model: sand fly count~type of village*number of applications + (1|trapsUID). Application 0 refers to the baseline, i.e., before any intervention.
(PDF)

S1 Table CL case burden between 2017–2019 for the 10 villages out of 410 with the highest number of CL cases in 2019 within Zagora province. Villages included in the study are highlighted in grey.
(PDF)

S2 Table The incidence rate ratio of the female sand fly counts in function of the application number (0, 1, 2, 3) and the village type (control, intervention) by sampling location (field, outdoor, indoor) based on the GLMM model: sand fly count ~ type of village*number of applications. (PDF)

S3 Table The odds ratio of the *Leishmania* spp. infection rate in function of the application (before versus after the application) and the village type (control, intervention) based on the GLMM model: *Leishmania* positivity~type of village*application + (1|TrapsUID). (PDF)

S1 Data Database containing sand fly counts per village, location, collection date, survey, and sex. (CSV)

S2 Data Database containing sand fly counts per village, location, collection date, survey, sex and species (*Phlebotomus alexandri*, *Phlebotomus longicuspis*, and *Phlebotomus papatasi*). (CSV)

S3 Data Database containing the *Leishmania* detection in sand flies based on the SL-RNA qPCR per village, location, and collection date. (CSV)
