# Supplementary material for: Pilot study to assess the impact of feed-through insecticide on the sand fly density in an endemic area of zoonotic cutaneous leishmaniasis in Morocco
Source: PLoS Negl Trop Dis. 2025 Dec 18;19(12):e0013767. doi: 10.1371/journal.pntd.0013767 (PMC12747434; doi:10.1371/journal.pntd.0013767)

SUPPORTING INFORMATION

Pilot study to assess the impact of feed-through insecticide on the sand fly density in an endemic area of zoonotic cutaneous leishmaniasis in Morocco

S1 Fig. Georeferenced pictures taken by the field teams during bait application.

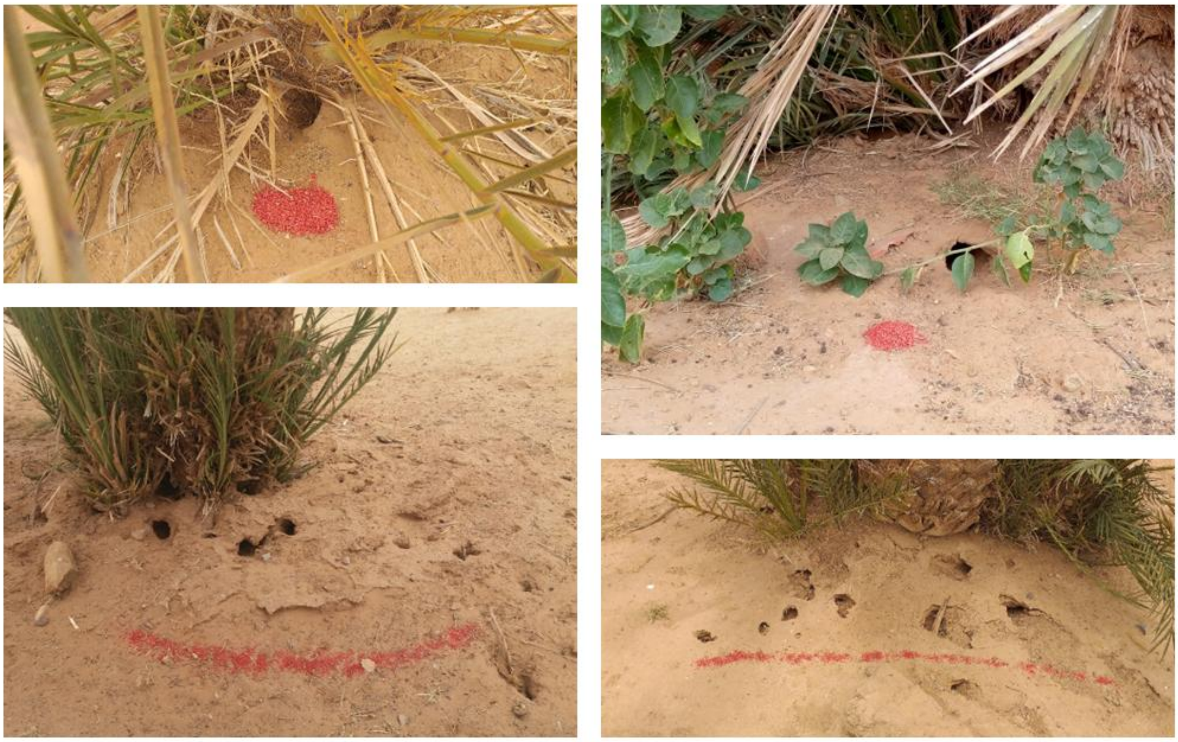

Supplement: S1 Fig — (PDF) [file pntd.0013767.s001.pdf]
