# Supplementary material for: Pilot study to assess the impact of feed-through insecticide on the sand fly density in an endemic area of zoonotic cutaneous leishmaniasis in Morocco
Source: PLoS Negl Trop Dis. 2025 Dec 18;19(12):e0013767. doi: 10.1371/journal.pntd.0013767 (PMC12747434; doi:10.1371/journal.pntd.0013767)

SUPPORTING INFORMATION

Pilot study to assess the impact of feed-through insecticide on the sand fly density in an endemic area of zoonotic cutaneous leishmaniasis in Morocco

S2 Fig. Predicted female sand fly density and their 95% confidence intervals in function of the type of village (control versus intervention) and application number (0, 1, 2, 3) by the sampling location (field, outdoor, indoor) and physiological status of the sand flies (fed, non-fed) based on the GLMM model: sand fly count ~ type of village*number of applications + (1|trapsUID). Application 0 refers to the baseline i.e., before any intervention.

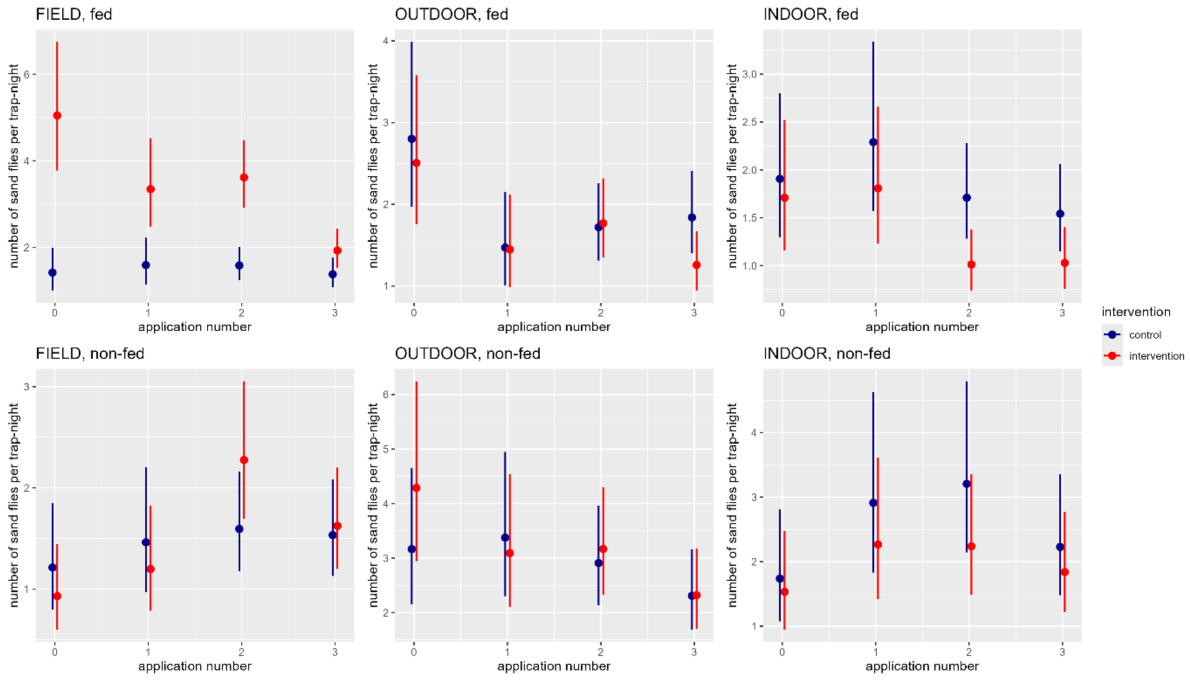

Supplement: S2 Fig — Application 0 refers to the baseline, i.e., before any intervention. (PDF) [file pntd.0013767.s002.pdf]
