# Supplementary material for: Pilot study to assess the impact of feed-through insecticide on the sand fly density in an endemic area of zoonotic cutaneous leishmaniasis in Morocco
Source: PLoS Negl Trop Dis. 2025 Dec 18;19(12):e0013767. doi: 10.1371/journal.pntd.0013767 (PMC12747434; doi:10.1371/journal.pntd.0013767)
Supplement: S1 Table — Villages included in the study are highlighted in grey. (PDF) [file pntd.0013767.s003.pdf]

SUPPORTING INFORMATION

Pilot study to assess the impact of feed-through insecticide on the sand fly density in an endemic area of zoonotic cutaneous leishmaniasis in Morocco

S1 Table. CL case burden between 2017-2019 for the 10 villages out of 410 with the highest number of CL cases in 2019 within Zagora province. Villages included in the study are highlighted in grey.

Locality (village)	CL 2017	CL 2018	CL 2019	Population 2019	Average annual incidence/1000 population	Intervention
Astour	17	145	69	1415	54,42	*NA*
Aid Mnad	32	44	57	3147	14,09	Intervention
Bni Zoli Centre	29	270	38	3039	36,96	*NA*
Oulad M'Saad	0	0	37	1528	8,07	Intervention
Tingdide	0	0	37	631	19,55	*NA*
Timsla	0	0	34	2320	4,89	Control
Zaite Tafardouste	49	88	33	1543	36,72	*NA*
Bouzergane	15	96	32	1590	29,98	Control
Oulad Slimane	2	20	31	1480	11,94	*NA*
Ighardaine	1	0	30	851	12,14	*NA*
