# Supplementary material for: Pilot study to assess the impact of feed-through insecticide on the sand fly density in an endemic area of zoonotic cutaneous leishmaniasis in Morocco
Source: PLoS Negl Trop Dis. 2025 Dec 18;19(12):e0013767. doi: 10.1371/journal.pntd.0013767 (PMC12747434; doi:10.1371/journal.pntd.0013767)
Supplement: S2 Table — (PDF) [file pntd.0013767.s004.pdf]

**SUPPORTING INFORMATION**

**Pilot study to assess the impact of feed-through insecticide on the sand fly density in an endemic area of zoonotic cutaneous leishmaniasis in Morocco**

**S2 Table.** The incidence rate ratio of the female sand fly counts in function of the application number (0, 1, 2, 3) and the village type (control, intervention) by sampling location (field, outdoor, indoor) based on the GLMM model: sand fly count ~ type of village*number of applications.

| *Phlebotomus papatasi* | | | | | | | | | |
|---|---|---|---|---|---|---|---|---|---|
| | **FIELD** | | | **OUTDOOR** | | | **INDOOR** | | |
| Predictors | Incidence Rate Ratio | 95CI | p | Incidence Rate Ratio | 95CI | p | Incidence Rate Ratio | 95CI | p |
| Intercept | 0.27 | 0.13 – 0.53 | **<0.001** | 1.72 | 1.07 – 2.93 | **0.035** | 0.43 | 0.24 – 0.82 | **0.008** |
| application 1 | 1.13 | 0.43 – 2.95 | 0.809 | 0.43 | 0.20 – 0.90 | **0.025** | 1.42 | 0.61 – 3.35 | 0.415 |
| application 2 | 0.34 | 0.13 – 0.89 | **0.030** | 0.32 | 0.16 – 0.60 | **0.001** | 1.04 | 0.48 – 2.20 | 0.922 |
| application 3 | 1.25 | 0.55 – 2.87 | 0.597 | 0.25 | 0.13 – 0.47 | **<0.001** | 1.25 | 0.59 – 2.63 | 0.558 |
| Intervention village | 0.94 | 0.35 – 2.50 | 0.897 | 1.66 | 0.82 – 3.36 | 0.156 | 1.69 | 0.73 – 3.94 | 0.219 |
| appl 1 x Intervention | 1.54 | 0.41 – 5.89 | 0.526 | 0.89 | 0.32 – 2.51 | 0.825 | 1.31 | 0.41 – 4.14 | 0.646 |
| appl 2 x Intervention | 7.66 | 2.19 – 27.47 | **0.002** | 1.29 | 0.53 – 3.14 | 0.578 | 1.11 | 0.40 – 3.08 | 0.848 |
| appl 3 x Intervention | 1.39 | 0.43 – 4.47 | 0.583 | 1.16 | 0.47 – 2.87 | 0.752 | 0.55 | 0.19 – 1.53 | 0.248 |

| *Phlebotomus alexandri* | | | | | | | | | |
|---|---|---|---|---|---|---|---|---|---|
| | **FIELD** | | | **OUTDOOR** | | | **INDOOR** | | |
| Predictors | Incidence Rate Ratio | 95CI | p | Incidence Rate Ratio | 95CI | p | Incidence Rate Ratio | 95CI | p |
| Intercept | 0.42 | 0.23 – 0.79 | **0.006** | 0.73 | 0.41 – 1.39 | 0.313 | 0.20 | 0.09 – 0.45 | **<0.001** |
| application 1 | 1.24 | 0.52 – 2.94 | 0.623 | 1.75 | 0.76 – 4.05 | 0.187 | 6.00 | 2.21 – 16.83 | **<0.001** |
| application 2 | 0.56 | 0.25 – 1.24 | 0.154 | 2.37 | 1.13 – 4.83 | **0.019** | 13.62 | 5.51 – 34.02 | **<0.001** |
| application 3 | 1.62 | 0.77 – 3.38 | 0.201 | 1.68 | 0.80 – 3.44 | 0.161 | 8.00 | 3.22 – 20.06 | **<0.001** |
| Intervention village | 0.12 | 0.03 – 0.43 | **0.003** | 0.43 | 0.17 – 1.08 | 0.073 | 0.83 | 0.26 – 2.67 | 0.758 |
| appl 1 x Intervention | 5.38 | 1.13 – 31.60 | **0.044** | 0.69 | 0.19 – 2.47 | 0.569 | 0.47 | 0.11 – 2.06 | 0.312 |
| appl 2 x Intervention | 30.36 | 7.31 – 162.23 | **<0.001** | 1.55 | 0.53 – 4.60 | 0.425 | 0.44 | 0.12 – 1.66 | 0.224 |
| appl 3 x Intervention | 5.04 | 1.23 – 26.56 | **0.035** | 1.56 | 0.53 – 4.68 | 0.420 | 0.56 | 0.15 – 2.12 | 0.391 |

| *Phlebotomus longicuspis* | | | | | | | | | |
|---|---|---|---|---|---|---|---|---|---|
| | **FIELD** | | | **OUTDOOR** | | | **INDOOR** | | |
| Predictors | Incidence Rate Ratio | CI | p | Incidence Rate Ratio | CI | p | Incidence Rate Ratio | CI | p |
| Intercept | 0.42 | 0.20 – 0.94 | **0.024** | 1.10 | 0.50 – 3.08 | 0.834 | 0.05 | 0.01 – 0.24 | **<0.001** |
| application 1 | 0.24 | 0.06 – 0.84 | **0.029** | 0.76 | 0.21 – 2.79 | 0.669 | 3.00 | 0.43 – 23.92 | 0.273 |
| application 2 | 0.40 | 0.14 – 1.06 | 0.070 | 0.14 | 0.04 – 0.43 | **0.001** | 5.17 | 0.90 – 32.39 | 0.064 |
| application 3 | 0.42 | 0.15 – 1.11 | 0.085 | 0.23 | 0.07 – 0.71 | **0.013** | 11.50 | 2.03 – 70.91 | **0.005** |
| Intervention village | 0.48 | 0.15 – 1.52 | 0.211 | 0.26 | 0.07 – 1.00 | **0.045** | 6.33 | 0.96 – 48.21 | 0.058 |
| appl 1 x Intervention | 4.86 | 0.85 – 29.04 | 0.078 | 1.55 | 0.23 – 10.35 | 0.645 | 0.68 | 0.05 – 8.45 | 0.767 |
| appl 2 x Intervention | 4.58 | 1.11 – 19.30 | **0.036** | 7.55 | 1.39 – 41.47 | **0.019** | 0.22 | 0.02 – 2.03 | 0.187 |
| appl 3 x Intervention | 4.66 | 1.13 – 19.53 | **0.033** | 5.89 | 1.12 – 31.20 | **0.035** | 0.11 | 0.01 – 0.98 | **0.049** |
