# Supplementary material for: Pilot study to assess the impact of feed-through insecticide on the sand fly density in an endemic area of zoonotic cutaneous leishmaniasis in Morocco
Source: PLoS Negl Trop Dis. 2025 Dec 18;19(12):e0013767. doi: 10.1371/journal.pntd.0013767 (PMC12747434; doi:10.1371/journal.pntd.0013767)
Supplement: S3 Table — (PDF) [file pntd.0013767.s005.pdf]

S3 Table. The odds ratio of the *Leishmania* spp. infection rate in function of the application (before versus after the application) and the village type (control, intervention) based on the GLMM model: *Leishmania* positivity ~ type of village*application + (1|TrapsUID).

	**OUTDOOR**		
**Predictors**	**Odds Ratio**	**CI**	**p**
(Intercept)	0.01	0.00 – 0.06	**<0.001**
Application	1.52	0.31 – 7.49	0.607
Intervention village	2.37	0.42 – 13.39	0.329
Application × Intervention village	0.31	0.04 – 2.50	0.275
Application: the data from surveys 3—6 were pooled i.e., surveys after the application of the feed-through insecticide.			
